# Effects of an Activity Tracker and App Intervention to Increase Physical Activity in Whole Families—The Step It Up Family Feasibility Study

**DOI:** 10.3390/ijerph17207655

**Published:** 2020-10-20

**Authors:** Stephanie Schoeppe, Jo Salmon, Susan L. Williams, Deborah Power, Stephanie Alley, Amanda L. Rebar, Melanie Hayman, Mitch J. Duncan, Corneel Vandelanotte

**Affiliations:** 1 Physical Activity Research Group, School of Health, Medical and Applied Sciences, Appleton Institute, Central Queensland University, Building 77, Bruce Highway, Rockhampton, QLD 4702, Australia; s.p.williams@cqu.edu.au (S.L.W.); d.a.power@cqu.edu.au (D.P.); s.alley@cqu.edu.au (S.A.); a.rebar@cqu.edu.au (A.L.R.); m.j.hayman@cqu.edu.au (M.H.); c.vandelanotte@cqu.edu.au (C.V.); 2 School of Exercise and Nutrition Sciences, Institute for Physical Activity and Nutrition (IPAN), Deakin University, 221 Burwood Highway, Burwood, Geelong, VIC 3125, Australia; jo.salmon@deakin.edu.au; 3 Priority Research Centre for Physical Activity and Nutrition, School of Medicine & Public Health, Faculty of Health and Medicine, The University of Newcastle, University Drive, Callaghan, Newcastle, NSW 2308, Australia; Mitch.Duncan@newcastle.edu.au

**Keywords:** family-centered, intervention, children, maternal, paternal, active, steps, smartphone, tablet, apps, fitness trackers, wearables

## Abstract

(1) Background: Interventions using activity trackers and smartphone apps have demonstrated their ability to increase physical activity in children and adults. However, they have not been tested in whole families. Further, few family-centered interventions have actively involved both parents and assessed physical activity effects separately for children, mothers and fathers. Objective: To examine the feasibility and short-term effects of an activity tracker and app intervention to increase physical activity in the whole family (children, mothers and fathers). (2) Methods: This was a single-arm feasibility study with pre-post intervention measures. Between 2017–2018, 40 families (58 children aged 6–10 years, 39 mothers, 33 fathers) participated in the 6-week *Step it Up Family* program in Queensland, Australia. Using commercial activity trackers combined with apps (Garmin Vivofit Jr for children, Vivofit 3 for adults; Garmin Australasia Pty Ltd., Sydney, Australia), the intervention included individual and family-level goal-setting, self-monitoring, performance feedback, family step challenges, family social support and modelling, weekly motivational text messages and an introductory session. Parent surveys were used to assess physical activity effects measured as pre-post intervention changes in moderate-to-vigorous physical activity (MVPA) in children, mothers and fathers. Objective Garmin activity tracker data was recorded to assess physical activity levels (steps, active minutes) during the intervention. (3) Results: Thirty-eight families completed the post intervention survey (95% retention). At post intervention, MVPA had increased in children by 58 min/day (boys: 54 min/day, girls: 62 min/day; all *p* < 0.001). In mothers, MVPA increased by 27 min/day (*p* < 0.001) and in fathers, it increased by 31 min/day (*p* < 0.001). The percentage of children meeting Australia’s physical activity guidelines for children (≥60 MVPA min/day) increased from 34% to 89% (*p* < 0.001). The percentage of mothers and fathers meeting Australia’s physical activity guidelines for adults (≥150 MVPA min/week) increased from 8% to 57% (*p* < 0.001) in mothers and from 21% to 68% (*p* < 0.001) in fathers. The percentage of families with ‘at least one child and both parents’ meeting the physical activity guidelines increased from 0% to 41% (*p* < 0.001). Objective activity tracker data recorded during the intervention showed that the mean (*SD*) number of active minutes per day in children was 82.1 (17.1). Further, the mean (*SD*) steps per day was 9590.7 (2425.3) in children, 7397.5 (1954.2) in mothers and 8161.7 (3370.3) in fathers. (4) Conclusions: Acknowledging the uncontrolled study design, the large pre-post changes in MVPA and rather high step counts recorded during the intervention suggest that an activity tracker and app intervention can increase physical activity in whole families. The *Step it Up Family* program warrants further efficacy testing in a larger, randomized controlled trial.

## 1. Introduction

In Australia, more than 80% of children aged 5–17 years do not get the recommended 60 min of moderate-to-vigorous physical activity (MVPA) a day [1] and 55% of adults do not achieve the recommended 150 min of moderate-to-vigorous physical activity a day [2]. Physical inactivity is a significant contributor to Australia’s high prevalence of overweight and obesity in children (25%) and adults (67%) [3]. Further, physical inactivity is a leading cause in the development of major chronic diseases such as cardiovascular disease, diabetes and cancer [4] and costs the Australian economy $13.8 billion a year [5]. Often, the physical inactivity epidemic starts in childhood, exacerbates in adolescence and continues throughout adulthood [6]. Given the large burden of disease associated with physical inactivity innovative approaches are needed that increase physical activity affordably in large numbers of children and adults. 

Family dynamics have the power to increase physical activity in children and adults simultaneously. Children are most active when both mothers *and* fathers support and model active behaviors through encouragement, praise and co-participation in outdoor play, sports and active recreation [7,8,9,10]. As such, it is important to actively involve both parents in physical activity interventions for families [8,11]. Furthermore, children often ask their parents to engage in active play and sports with them [12]. As such, children can be a key driver for physical activity participation in their parents [13]. However, many families live a “couch potato” lifestyle and need motivation for an active lifestyle [14]. 

Activity trackers combined with smartphone applications (apps) have become popular self-monitoring systems to help people in becoming more active [12,15,16,17]. In 2018, 2.1 million Australians used activity trackers and 75% of Australians used apps [18,19]. Notably, parents are early adopters of wearable activity trackers and the number one motivating reason for using these devices is ‘improving health’ [20]. Recent systematic reviews [21,22,23] have shown that interventions using activity trackers combined with apps can effectively increase physical activity in children and adults. The popularity of activity trackers and apps in the general population and their proven efficacy in both children and adults suggest that this technology has great potential to promote physical activity in families. 

To our knowledge, no physical activity interventions using commercial activity trackers in combination with apps have actively engaged whole families. Moreover, there are several other research gaps pertaining to family-centered physical activity interventions. Firstly, previous pediatric physical activity and health interventions in families have almost exclusively engaged mothers [11,24], with fathers representing a mere 6% of parent participants [11,24]. This is often because they have not been specifically invited to participate (typically ‘parents in general’ are invited and only the mother participates) [25]. Failing to engage fathers as important role models of physical activity is particularly unfortunate given that fathers’ emotional bond with their children primarily develops through active play [26]. Secondly, the idea that children support their parents to become more physically active has rarely been implemented in previous family-centered interventions [13,27] which have usually focused on parents (mostly only the mother) helping their children become more active [28,29]. Thirdly, maternal and paternal outcomes have rarely been assessed separately in previous family-centered physical activity interventions [26]. This is important to identify reciprocal influences between children, mothers and fathers [26].

### Objective

The *Step it Up Family* program was designed to address these aforementioned research gaps. It aimed to examine the short-term effects of an activity tracker- and app intervention to increase physical activity in the whole family (children, mothers and fathers). 

## 2. Methods

### 2.1. Study Design 

The *Step it Up Family* program was a single-arm feasibility study with pre-post intervention measures conducted at Central Queensland University in Rockhampton, Australia. The design was appropriate for investigating the feasibility and potential effectiveness of an innovative intervention approach to increase physical activity levels in wholes families (i.e., children aged 6–10 years, mothers and fathers). This feasibility study was implemented in preparation for a more costly, large-scale randomized controlled trial to assess the long-term intervention efficacy of the Step it Up Family program. Ethical approval for the study was received from the Central Queensland University Human Ethics Committee in May 2017 (H17/03-041). Written informed participant consent was obtained online from both mothers and fathers and one parent provided the participant consent on behalf of the participating children.

### 2.2. Participants

Between May 2017 and October 2018, 40 families living in Queensland Australia (mostly Central and South East Queensland) were recruited into the *Step it Up Family* program. Recruitment was paused during the Australian summer school holiday period December 2017–January 2018. The study examined the active engagement of both mothers *and* fathers in a family-centered physical activity intervention, from implementation to evaluation. Family eligibility required that mothers and fathers older than 18 years (including step-mothers/fathers and female/male guardians) and at least one child aged 6–10 years participate in the study. Children aged 6–10 years were targeted as this age range is crucial for forming physical activity behaviors, spending time with parents and being influenced by parental social support and role modelling [10]. Children younger than five years were not enrolled due to limited ability to comprehend activity tracker and app features. Children older than 10 years were not enrolled as (pre)adolescents are more influenced by physical activity role modelling and support from peers rather than parents [30]. However, non-enrolled siblings could still receive an activity tracker, ensuring no child in the family was left out which may have undermined the positive family dynamics (e.g., family step challenges). Since the study focused on active engagement of both mothers *and* fathers in a family-centered intervention, single parent and same sex parent families were initially excluded. However, family eligibility was later relaxed in order to expedite the recruitment target of 40 families. Hence, 8 (20%) single parent families (7 mother-child dyads, 1 father-child dyad) were included. 

Other study inclusion criteria included: all family members spoke and read English; all family members lived together in one household; all enrolled parents had access to the Internet and a smartphone or tablet; the child had not previously used an activity tracker (e.g., pedometer, Garmin, Fitbit, Apple Watch) to increase physical activity and all family members could safely increase physical activity. Further, children and parents had to be ‘insufficiently active’ at baseline which was assessed by one parent reporting child and parent physical activity levels in an online screening survey. Insufficiently active was defined as not meeting Australia’s Physical Activity Guidelines (children: <60 daily minutes of moderate to vigorous physical activity; adults: <150 weekly minutes of moderate to vigorous physical activity) [1]. Finally, the mother was not pregnant at the time of recruitment, as pregnancy can affect physical activity levels and motivation [31]. 

### 2.3. The Step It Up Family Intervention

The *Step it Up Family* program was designed to mobilize a whole family (children aged 6–10 years, mothers and fathers) to become more physically active. The intervention incorporated evidence-based health behavior change techniques (e.g., goal setting, self-monitoring, performance feedback, social support and role modelling) [32,33] tailored to physical activity behavior. Further, the intervention targeted core constructs of social cognitive theory (e.g., self-efficacy, modelling, reinforcement) [34] and self-determination theory (i.e., autonomy, competence, relatedness) [35] (Table 1). 

The core intervention components are detailed in Table 1 and the intervention materials are presented in the Supplementary File S1. Overall, the 6-week intervention included an introductory session (delivered face-to-face or via telephone), family resources (i.e., activity trackers and apps, family step challenge log poster, informational leaflets) and motivational and educational text messages (sent 3x per week to parents). Primarily, *Step it Up Family* was an activity tracker and app intervention. Children, mothers and fathers received age-specific activity trackers combined with apps (Garmin Vivofit Jr for children, Garmin Vivofit 3 for adults; Garmin Australasia Pty Ltd., Sydney, Australia) to motivate themselves and each other to increase daily and weekly steps, as well as active minutes. The Garmin Vivofit Jr activity trackers we utilized in children have previously demonstrated high feasibility for monitoring physical activity in children aged 4–10 years [36]. Further, the Garmin Vivofit activity trackers we utilized in parents have shown acceptable validity for monitoring steps in adult populations [37]. The detailed design and features of the activity trackers and apps used in the intervention are presented in the Supplementary File S1. Briefly, the Garmin Vivofit Jr activity tracker had a child-friendly design as it displayed children’s steps and progress to reaching the recommended 60 min of physical activity, it was waterproof and its battery lasted one year. The corresponding Vivofit Jr app (installed and controlled via parents’ smartphone/tablet) displayed the steps of all enrolled family members in a family leaderboard. Additionally, the Garmin Vivofit Jr activity tracker and app had other fun features children could opt to use (e.g., bright color band, watch, personal name and animal images on display, virtual coins as rewards, virtual adventure trail). Using the activity trackers and apps daily for six weeks, both children and parents were instructed during the introductory session to set individual and family-level graded step goals, self-monitor steps and active minutes individually and as a family, conduct family step challenges and use the family leaderboard shown in the app to monitor individual and family progress. Children, mothers and fathers attended the face-to-face delivered introductory session together to actively involve the whole family from the start. When the introductory session was delivered via telephone to families living further away, all participating family members were asked to be present. However, this could not be controlled by the project officer. In the introductory session, the family members were asked to become physical activity role models and support each other in becoming more physically active individually and as a family. For this, both children and parents were given examples of goal-setting and physical activity social support and modelling. 

### 2.4. Procedures

Recruitment, intervention delivery and data collection were carried out by a trained research officer who was employed at Central Queensland University, Physical Activity Research Group in Rockhampton, Australia. The research officer was well connected to the Yeppoon and Rockhampton communities and experienced with family-centered intervention research. Another research assistant helped with the recruitment process. The families were recruited through multiple channels: (1) one Facebook advertisement, (2) 11 Facebook groups (e.g., Yeppoon and Rockhampton Regional Councils, Yeppoon Families), (3) 78 local organizations (e.g., schools, kindergartens, youth and community organizations, sporting facilities, businesses, politicians’ office), (4) six local media (newspaper, radio) and (5) word-of-mouth. An online screening survey was used to determine family eligibility. One parent (91% mothers) completed the screening survey for the family and if potentially eligible, further confirmation was sought via telephone interview. After recruitment, parents received an email with a link to an online participant consent form and baseline survey. The participant consent form and all online survey assessments (baseline, post intervention) were completed by mothers and fathers, respectively. In addition, one parent (93% mothers) completed the participant consent and survey questions on behalf of the participating children (as children younger than 10 years do not provide reliable and valid survey data). Upon completion of consent form and baseline survey by both parents, families received the introductory session and family resources (i.e., activity trackers and apps, family step challenge log poster, informational leaflets). To accommodate families’ schedules and geographic location, the delivery mode of the introductory session was organized conveniently for each family. This included delivery via telephone (35% of families) or face-to-face (65% of families) at Central Queensland University Rockhampton Campus, a public playground or the family home. Furthermore, the introductory session was scheduled at families preferred time of the day and lasted for approximately 60 min. When the introductory session was delivered via telephone, families were posted beforehand the family resources including instructions on how to download the apps to their smartphones/tablets. The screening, baseline and post intervention survey data were collected online using SurveyMonkey software. Families received up to four shopping vouchers (3 × 20 AUD voucher, 1 × 30 AUD voucher) as a compensation for their time and to encourage retention and minimize Garmin activity tracker and data loss. The first two 20 AUD vouchers were handed out at the face-to-face introductory session (or posted with the family resources if the introductory session was delivered via telephone), provided both parents attended. If one parent attended, one voucher was handed out. A third voucher was posted mid-intervention if all family members had recorded steps and active minutes via the Garmin apps (daily and weekly recordings were downloaded by research staff during the intervention through the access of participants app accounts). The fourth voucher (30 AUD voucher) was posted at the end of the intervention, upon completion of the post intervention survey and return of the Garmin activity trackers. 

### 2.5. Measures

Parent (maternal, paternal) survey data were used to separately assess outcomes in children, mothers and fathers. Sociodemographic information assessed included sex, age, work status (employed: full-time, part-time, casual; unemployed: home duties, student, retired), education (in years), ethnicity (Caucasian, African, Asian; Aboriginal, Torres Strait Islanders and Pacific Islanders; Other) and families’ geographic location (major city, regional, remote, very remote). This paper presents the intervention feasibility in terms of family recruitment and retention, intervention delivery and fidelity, intervention engagement measured objectively through activity tracker and app usage and effects on physical activity levels in children, mothers and fathers and at the family level. More detailed process evaluation data on intervention feasibility in terms of acceptability and perceived usefulness of the intervention components were collected in children, mothers and fathers through parent surveys and semi-structured interviews conducted via telephone/Skype (Skype Technologies, Luxembourg City, Luxembourg) at post intervention. These study feasibility outcomes will be reported in detail elsewhere (Schoeppe et al., unpublished data). 

#### 2.5.1. Process Measures

Family recruitment was determined through (1) the online screening survey completed by one parent to assess family eligibility and if eligible, (2) the online participant consent form completed by both parents. Participation was measured as the proportion of families commencing the intervention after providing consent. Retention was measured as the proportion of families completing the post intervention survey. Furthermore, intervention delivery and fidelity were assessed using research staff records of procedures relating to recruitment, intervention delivery and data collection, in particular, modifications to the study protocol after commencement of the study.

#### 2.5.2. Parent-Reported Physical Activity in Children Pre vs. Post Intervention

The parental proxy questionnaire of the Children’s Leisure Activity Study Survey (CLASS) [38] was used to assess moderate-to-vigorous physical activity (MVPA) in children aged 6–10 years pre versus post intervention. The CLASS comprises a checklist of 30 physical activities. Of these, 18 activities are classified as moderate intensity: baseball or softball, bicycling, dance, downball, gymnastics, household chores, physical education class, playground equipment, playing in playhouse, playing with pets, school sport class, scooter, skateboard, trampoline, travel to school by walking, travel to school by bicycling, walking for exercise and walking the dog. Twelve activities are classified as vigorous: aerobics, Australian-rules football, basketball, jogging or running, netball, rollerblading, skipping, soccer, swimming for fun, swimming laps, playing tag or chasey and playing tennis or bat tennis. For each physical activity in the checklist, parents were asked to circle yes or no, indicating whether their child participates in that activity during a typical week (Monday to Friday) and during a typical weekend (Saturday and Sunday). A ‘typical week’ was defined as being during the current school term, not including school holidays. If they circled yes, parents were asked to report the frequency of the activity (how many times Monday–Friday and Saturday–Sunday) and the total time their child spent in that activity (minutes or hours Monday–Friday and Saturday–Sunday). Mean daily minutes of MVPA was calculated by summing the minutes per week spent in moderate and vigorous physical activity and then averaging per day (divided by seven) [38]. The CLASS parental proxy questionnaire has demonstrated acceptable reliability (ICC = 0.69 and 0.74 for MVPA frequency and duration, respectively) [38]. Using the CLASS derived mean daily minutes of MVPA, children were classified as meeting Australia’s Physical Activity Guidelines for children aged 5–17 years (≥60 min of MVPA per day) or not (<60 min of MVPA per day).

#### 2.5.3. Self-Reported Physical Activity in Mothers and Fathers Pre vs. Post Intervention

The Active Australia Survey (AAS) [39] measure was used to assess MVPA separately in mothers and fathers pre versus post intervention. The AAS measures the duration and frequency of recreational and transport-related walking, as well as moderate and vigorous intensity physical activity during leisure-time [39]. Firstly, weekly minutes of MVPA was calculated by summing the time spent in walking, moderate physical activity and vigorous physical activity (weighted by two) as specified in the Active Australia Survey scoring guidelines [39]. Secondly, mean daily minutes of MVPA was calculated by averaging weekly minutes of MVPA per day (divided by seven). The AAS has demonstrated acceptable reliability (ICC = 0.64) [39] and criterion validity (*r* = 0.61) when compared to an objective accelerometer measure [40]. Using the AAS derived weekly minutes of MVPA, mothers and fathers were classified as meeting Australia’s Physical Activity Guidelines for adults aged 18–64 years (≥150 min of MVPA per week) or not (<150 min of MVPA per week).

#### 2.5.4. Objective Physical Activity in Children, Mothers and Fathers During the Intervention

Objective Garmin activity tracker and app usage data was collected to assess the physical activity levels in children, mothers and fathers during the intervention. Throughout the 6-week intervention, families were required to regularly sync their activity tracker data on the Garmin Vivofit Jr (child) app and Garmin Connect (parent) app. Once a week, the research officer logged into the child and parent app accounts to extract daily step counts and active minutes. Using this activity tracker data, we calculated mean number of recording days, mean steps per day and mean active minutes per day. Active minutes per day were calculated for children only, as active minutes data from parents were not available due to technical difficulties.

### 2.6. Sample Size

This was a small-scale, single-arm feasibility study to examine intervention feasibility and short-term effects on physical activity levels. Based on pragmatic considerations of the feasibility of ‘whole family’ recruitment and available resources no formal sample size calculation was performed prospectively. We aimed for 40 families participating in the intervention to test the feasibility and short-term effects on physical activity when delivering this intervention approach in ‘whole families’ (i.e., at least one child aged 6–10 years, mothers and fathers). However, post-hoc power calculations were performed in PROC POWER in SAS version 9.4 (SAS, Cary, North Carolina, USA) to calculate power for the MVPA outcomes based on the statistical test used in the analyses. The power was about 99% indicating that the study was sufficiently powered to detect differences in MVPA between pre and post intervention. 

### 2.7. Statistical Analyses

Paired sample *t*-tests were used to compare pre-post intervention differences in mean daily minutes spent in MVPA (continuous outcome variable) in children, mothers and fathers. McNemar’s test was used to compare pre-post intervention differences in the percentage of children, mothers and fathers who were meeting the Australian physical activity guidelines or not (dichotomous outcome variable). McNemar’s test was further used to compare pre-post intervention differences in the percentage of families with (1) ‘at least one child and one parent’ meeting the Australian physical activity guidelines and those with (2) ‘at least one child and both parents’ meeting the physical activity guidelines (dichotomous outcome variables). Analyses were performed for participants with complete data (i.e., pre and post intervention) and as intention-to-treat analyses with baseline scores carried through to post intervention (i.e., a conservative intention-to-treat approach) for participants who did not complete the post intervention survey. Analyses were performed in IBM SPSS Statistics version 26.0 (IBM Australia Ltd., Sydney, Australia) using an alpha level of 0.05.

## 3. Results

### 3.1. Recruitment, Participation and Retention

Eighty-one families completed the screening survey of which 76 families provided written parental and child consent to participate in the study. Of these, 36 families were excluded because they either did not complete the baseline survey, did not set up their activity trackers and app software or withdrew from the study prior to intervention commencement. In total, 40 families including 58 children (50% girls), 39 (98%) mothers and 33 (83%) fathers participated in the *Step it Up Family* program. Of these, 38 families completed the post intervention survey (95% overall family retention; 90% children, 95% mothers, 88% fathers). Complete pre-post intervention outcome data on physical activity levels were obtained from 44 (76%) of children, 37 (95%) of mothers and 28 (85%) of fathers. There were no significant differences in baseline characteristics between those lost to follow-up and those retained (all *p* > 0.05).

### 3.2. Baseline Data

The majority of families (80%) were located in a regional area; fewer families (20%) lived in a major city. Most families (58%) had one child enrolled in the program, other families (40%) had two children enrolled and one family had three children enrolled. Baseline statistics of the participants are presented in Table 2. The mean (*SD*) ages of children, mothers and fathers were 8.0 (1.5) years, 37.8 (4.3) years and 41.2 (6.1) years, respectively. Of the parents, 71% had 13+ years of education, 82% were employed and 99% were Caucasian. Mean daily minutes of MVPA were 56.1 in children, 8.6 in mothers and 10.4 in fathers. Only 33% of children, 8% of mothers and 19% of fathers were meeting Australia’s physical activity guidelines of ≥60 min/day of MVPA for children and ≥150 min/week of MVPA for adults, respectively. 

### 3.3. Delivery and Fidelity of the Intervention

The initial intervention protocol was that the introductory session is delivered face-to-face and the child(ren), mother and father were required to attend the introductory session together in order to actively involve the whole family from the start. However, the face-to-face introductory session was difficult to schedule for some families (due to time constraints or families living further away). Those families were offered to receive the introductory session via telephone. Using this modified procedure, it was difficult to ensure that all enrolled family members joined the introductory session. However, offering more flexibility to families in the delivery mode of the introductory session helped retain families at the beginning of the study and commence the otherwise online-delivered 6-week intervention. In the end, both delivery modes (face-to-face, telephone) proofed suitable to explain families the intervention components and set up their activity trackers and app accounts. The delivery of the motivational and educational text messages sent 3× per week to parents’ smartphone throughout the intervention worked smoothly and therefore was delivered consistently to all families. In some children, the child sized wrist band of the Garmin Vivofit Jr activity tracker was too tight. These children were happy to wear their Vivofit Jr activity tracker with an adult sized wrist band (choice of black or white color), the same Garmin wrist band model their parents wore. Some families found the baseline and post intervention questionnaires too long, particularly when one parent also had to complete questions on behalf of the children. However, the baseline and post intervention questionnaires were not amended during the study to ensure data collection was consistent for all families. To increase families’ compliance with completing the post intervention survey the value of the shopping voucher mailed post intervention was increased from $20 to $30. The most significant deviation from the study protocol was extending family eligibility for the program to single-parent families in order to expediate recruitment and reach the target sample of 40 families. As a result, eight single parent families (7 mother-child dyads, 1 father-child dyad) participated in the Step it Up Family program. Since it was a feasibility study, we accepted this study protocol modification later in the recruitment period.

### 3.4. Pre-Post Intervention Effects in Children, Mothers and Fathers

The pre-post intervention physical activity changes in children, mothers and fathers are presented in Table 3. Significant intervention effects were detected for physical activity levels in children, mothers and fathers. Overall, children increased their MVPA by 58 min/day (*p* < 0.001). In boys, MVPA increased by 54 min/day (*p* < 0.001) and in girls, it increased by 62 min/day (*p* < 0.001). Parents increased their MVPA by 29 min/day (*p* < 0.001). Mothers’ MVPA increased by 27 min/day (*p* < 0.001) and fathers’ MVPA increased by 31 min/day (*p* < 0.001). When the analyses were rerun as intention-to-treat analyses, the intervention effects remained significant in children, mothers and fathers (Table 3). Given that children’s baseline MVPA was relatively high (56 min/day), we further assessed the pre-post intervention physical activity changes in children (using complete pre-post MVPA data) with low MVPA (1–30.99 mean min/day), medium MVPA (31–63.99 mean min/day) and high MVPA (≥64 mean min/day) at baseline. In children with low MVPA at baseline, MVPA increased by 83 min/day (*p* < 0.001; *n* = 15) at post intervention. In children with medium MVPA at baseline, MVPA increased by 38 min/day (*p* = 0.010; *n* = 14) at post intervention and in children with high MVPA at baseline, it increased by 52 min/day (*p* = 0.002; *n* = 15) at post intervention.

**Figure 1:**
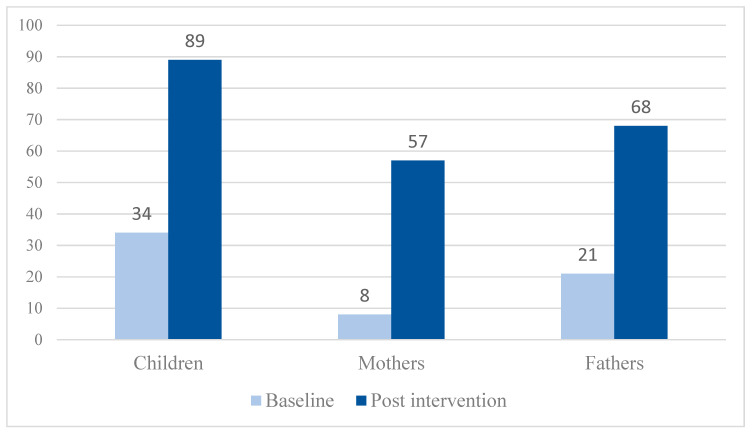
Percentage of children, mothers and fathers meeting Australia’s physical activity guidelines at baseline versus post intervention.

The percentage of children meeting Australia’s physical activity guidelines for children aged 5–17 years (≥60 MVPA min/day) increased from 34% at baseline to 89% at post intervention (*p* < 0.001) (Figure 1; based on sample including participants with pre and post intervention data). The percentage of mothers meeting Australia’s physical activity guidelines for adults aged 18–64 years (≥150 MVPA min/week) increased from 8% at baseline to 57% at post intervention (*p* < 0.001). In fathers, it increased from 21% at baseline to 68% at post intervention (*p* < 0.001). 

### 3.5. Pre-Post Intervention Effects at the Family Level

Physical activity also increased significantly at the family level. The percentage of families with ‘at least one child and one parent’ meeting the Australian physical activity guidelines increased from 7% at baseline to 72% at post intervention (*p* < 0.001). Furthermore, the percentage of families with ‘at least one child and both parents’ meeting the physical activity guidelines increased from 0% at baseline to 41% at post intervention (*p* < 0.001).

### 3.6. Physical Activity Levels in Children, Mothers and Fathers during the Intervention

**Figure 2:**
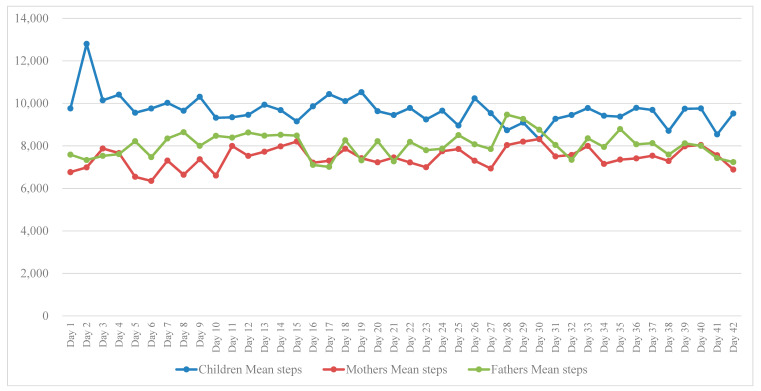
Mean steps per day in children, mothers and fathers during the 6-week (42-day) intervention.

The objective Garmin activity tracker data showed that during the 42-day intervention period the mean (*SD*) number of recording days using the activity tracker was 36.5 (8.3) in children, 38.5 (7.7) in mothers and 38.2 (8.8) in fathers. The mean (*SD*) active minutes per day in children was 82.1 (17.1). Further, the mean (*SD*) steps per day was 9590.7 (2425.3) in children, 7397.5 (1954.2) in mothers and 8161.7 (3370.3) in fathers. The mean steps per day in children, mothers and fathers recorded objectively with the activity tracker during the 6-week (42-day) intervention are also presented in Figure 2.

## 4. Discussion

This study tested the short-term effects of an activity tracker- and app intervention to increase physical activity in the whole family. Significant increases in physical activity were detected in children, mothers and fathers at post intervention. The physical activity increases are remarkably large and clinically important [41,42] with children getting nearly 60 min and both parents nearly 30 min more MVPA a day. The large pre-post changes in physical activity are supported by objective activity tracker data recorded during the intervention which showed that mean steps per day were 9591 in children, 7398 in mothers and 8162 in fathers. It must be acknowledged though that the *Step it Up Family* intervention was a single-arm feasibility study with pre-post survey measures. As such, the findings must be interpreted with caution as the intervention requires further testing in a randomized controlled trial using objective physical activity measurement. Nevertheless, the significant physical activity increases observed in children, mothers and fathers are encouraging and may demonstrate the importance and health potential of actively engaging both mothers *and* fathers in a family-centered physical activity intervention. This has rarely been implemented in previous pediatric physical activity and health interventions in families which have typically engaged solely mothers [11,24,29]. 

The family is a unit where children and parents influence each other through their physical activity modelling and social support [12,43]. Also, children like to copy, impress and compete with their parents and siblings [12]. These powerful family dynamics, particularly the bi-directional relationship between child and parent physical activity behavior [43], were harnessed in the *Step it Up Family* program when mobilizing the whole family to increase their steps. Our physical activity findings are notable given the paucity of family physical activity interventions targeting children and parents equally for increasing physical activity levels [29,44]. Most previous family interventions in this field [29] have engaged parents in the intervention with the primary outcome being physical activity levels in children. In contrast, the primary outcome in *Step it Up Family* was physical activity levels in children, mothers and fathers.

The recruitment of whole families comprising children, mothers and fathers into the *Step it Up Family* program took a long time (i.e., 16 months to recruit 40 families) when implemented on a small project budget by one part-time employed research officer. However, once recruited, families’ retention in the program was high (95% overall family retention; 90% children, 95% mothers, 88% fathers). Interestingly, our study was successful in screening and recruiting inactive families as demonstrated by the small proportions of children (33%), mothers (8%) and fathers (19%) meeting Australia’s physical activity guidelines at baseline. Recruiting and retaining the physically inactive, hard-to-reach population into physical activity interventions is a well-known challenge for physical activity researchers [45]. 

Compared to most previous family-centered interventions [29,44], the *Step it Up Family* program was very ‘minimal’ in that beyond the introductory session it did not require participants to attend sessions. Families had to attend only one introductory session (face-to-face or via telephone) and were only contacted remotely (three text messages per week) from the project team throughout the intervention. Beyond this, families were free to decide how much they wanted to engage with the activity trackers and apps to complete the other intervention components. The minimal face-to-face commitment required from parents may have suited the active involvement of both mothers and fathers in the intervention. Since time is short in families [46], our predominantly online delivered activity tracker and app intervention may have facilitated the implementation of a physical activity program that involved both parents and reached into the home. Few previous family physical activity interventions have demonstrated this [24,26]. Interestingly, the objective Garmin activity tracker data in our study showed that the families highly engaged with the activity trackers, as demonstrated by the high number of recording days in children (37 days), mothers (39 days) and father (38 days) during the 42-day intervention.

The *Step it Up Family* program incorporated proven behavior change techniques [32,33] and core constructs of social cognitive theory [34], as for example, ‘social support, physical activity modelling and reinforcement’ between children and both parents. Through the family step challenges, children, mothers and fathers were asked to become ‘agents of change’ in their families to help each other become more active. The significant physical activity increases observed in mothers and fathers may be attributed to the fact that both parents were instructed and encouraged to role model active behaviors to their children. Similarly, children were asked to monitor their parents progress in achieving their step goals and support them to become more active. This reciprocal reinforcement between family members is likely to have acted as a source of motivation to become more active and it is particularly important when forming new physical activity behaviors [29,34]. Interestingly, telephone/Skype interviews conducted with Step it Up Families after the intervention (unpublished data) revealed that parents appreciated the program’s opportunity for connecting as a family through engaging in co-physical activities and doing the family step challenges together. This has appealed to families and is in line with previous family-centered interventions [13,27,29] suggesting that spending quality time as a family through participation in co-physical activities can be an effective intervention approach. 

### 4.1. Strengths and Limitations

This study represents an important contribution to the field of mobile health and family physical activity interventions. Few family-centered interventions have been delivered online using wearable activity trackers and smartphone apps [29] and to our knowledge, no study has yet detected significant physical activity improvements in children, mothers *and* fathers. The *Step it Up Family* program also demonstrated the feasibility of engaging a whole family in an activity tracker and app intervention, as we were able to retain children and both parents in the program. The program addressed many of the aforementioned research gaps in family physical activity interventions [11,24,26,28]. Other strengths of this study include the use of proven health behavior change techniques and theories, intention-to-treat analysis, validated pre-post physical activity measures, objective physical activity assessment during the intervention and study outcome measurement in children, mothers and fathers. The study also has several limitations that need to be noted. Despite its innovation and promising findings, results from this study are limited by the lack of a control group, the reliance on solely parent/self-reported physical activity measurement (which is prone to overreporting and social desirability bias), a relatively small sample size (*n* = 40 families comprising 130 study participants) and a short intervention period (6 weeks). 

### 4.2. Recommendations for Future Studies

Notwithstanding its methodological limitations, the intervention effects from this feasibility study are encouraging and warrant further investigation of the long-term efficacy of the *Step it Up Family* program in a larger sample, using the more rigorous randomized controlled trial design, objective physical activity measurement (e.g., by accelerometry) and a longer intervention period with multiple follow-ups. Furthermore, as a public health intervention aiming to increase physical activity at the population level, it is important to test the effectiveness of this intervention approach in the ‘real world’ where no restrictions apply to family eligibility, study participants’ activity levels at baseline, activity tracker/app brands and controlled trial conditions. For example, an ecological trial of the *Step it Up Family* program would provide ‘real world’ (i.e., translational) information about how an activity tracker and smartphone app intervention works in families. This ecological approach has been largely overlooked in the public health field [47]. Effectiveness outcomes (i.e., intervention uptake, engagement, retention, behavior change) gathered from an ecological trial would provide important evidence irrespective of the efficacy outcomes from a randomized controlled trial. Established mass-reach community programs (e.g., 10,000 Steps Australia Program, National Heart Foundation of Australia Walking Program) already use activity trackers and apps to improve physical activity in the ‘real world’ [47,48]. But surprisingly, no study has yet determined their usability, efficacy and effectiveness for increasing physical activity in families. 

## 5. Conclusions

Acknowledging the uncontrolled study design, the large pre-post intervention changes in MVPA and rather high step counts recorded during the intervention suggest that an activity tracker and app intervention can increase physical activity in whole families in the short-term. An online delivered physical activity program that recruits and engages whole families and achieves clinically significant increases in physical activity levels in children, mothers *and* fathers may be an effective, scalable intervention for population health. The *Step it Up Family* program warrants further testing in a larger, randomized controlled trial to determine its long-term efficacy.

## Figures and Tables

**Table 1:** Description of Intervention Components in the Step it Up Family Program.

Intervention Component	Description	Incorporated Behavior Change Techniques ^a^	Targeted Behavior Change Theory Mediators ^b^
Introductory session	Child and parent activity trackers were set up and the respective apps downloaded on parents’ smartphone/iPad. All intervention components were explained. Children, mothers and fathers were educated on the importance of physical activity for health, were presented with the Australian physical activity guidelines for children and adults and given examples of how to model and support each other to become more physically active. The introductory session was delivered by a project officer either face-to-face (in 65% of families) at Central Queensland University, a public playground or families’ homes or via telephone in families who lived further way (in 35% of families).	Provide instructions Provide information about behavior-health link Prompt identification as role model Plan social support	Goals (SCT) Outcome expectations (SCT) Social support/relatedness (SCT/SDT) Modelling (SCT)
Family resources	*Garmin Vivofit activity trackers:* Children received the Garmin Vivofit Junior activity tracker. Mothers and fathers received the Garmin Vivofit 3 activity tracker for adults. *Garmin apps:* The Garmin Vivofit Junior app for children and the Garmin Connect app for adults were installed on mothers’ and fathers’ smartphone/iPad. *Family step challenge log poster:* During the introductory session, families received a family step challenge log poster in A3 poster format together with magnets to stick on the fridge. Children and parents were encouraged to log their daily and weekly individual and family-level steps in the log poster. *Informational resources:* Families received leaflets including the Australian Physical Activity Guidelines, information and maps of local walking tracks, 50 tips for family-based physical activities.	Goal-setting Set graded tasks Self-monitoring Performance feedback Provide instructions Provide contingent rewards Provide opportunities for social comparison	Goals (SCT) Autonomy (SDT) Self-efficacy/perceived competence (SCT/ SDT) Outcome expectations (SCT) Social support/relatedness (SCT/SDT)
Motivational and educational text messages	Motivational and educational text messages were sent 3 times per week to parents’ smartphone to provide families tips for goal-setting and (co-) physical activities. Emphasis was on how children, mothers and fathers can support and model active behaviors.	Provide instructions Prompt intentions formation Set graded tasks Prompt specific goal-setting Prompt practice Provide general encouragement Prompt identification as role model Plan social support	Goals (SCT) Autonomy (SDT) Social support/relatedness (SCT/SDT) Modelling (SCT)
Setting individual and family goals	Setting individual and family goals for being more active daily and weekly. Individual: e.g., 10,000 steps per day, active time per day (60 min children, 30 min adults). Family: e.g., 200,000 steps per week, 60 min of family active recreation on weekend days.	Goal-setting	Goals (SCT) Autonomy (SDT)
Setting graded achievable goals	Gradually increasing daily and weekly goals for being active. Individual: e.g., 500 more steps/day the next week, increase daily outdoor activity by 5 min. Family: e.g., 20,000 more steps next week, increase weekly family active recreation by 20 min.	Set graded tasks	Goals (SCT) Autonomy (SDT)
Self-monitoring	Monitoring step counts and active minutes spent in light, moderate and vigorous physical activity using the activity trackers combined with apps.	Self-monitoring Performance feedback	Autonomy (SDT) Self-efficacy/perceived competence (SCT/ SDT)
Family leaderboard	Sharing physical activity levels between children, mother and father via a family leaderboard shown on the app which displayed who had the ‘highest step counts’ and ‘most active minutes.’	Performance feedback Provide opportunities for social comparison Provide contingent rewards	Social support/relatedness (SCT/SDT) Outcome expectations (SCT)
Family step challenges	Families completed daily and weekly family step challenges to energies children, mothers and fathers to support each other and become physical activity role models for each other. Firstly, children, mothers and fathers challenged each other to get the ‘highest step counts’ and ‘most active minutes’ daily and weekly (beat family members’ activity goals). Secondly, families pursued weekly challenges to achieve ‘higher step counts’ and ‘more active minutes’ each week together as a family (reach activity goals together as a family).	Prompt identification as role model Plan social support Model or demonstrate the behavior Provide opportunities for social comparison Provide contingent rewards	Goals (SCT) Autonomy (SDT) Self-efficacy/perceived competence (SCT/ SDT) Outcome expectations (SCT) Social support/relatedness (SCT/SDT) Modelling (SCT) Reinforcement (SCT)

^a^ Health Behavior Change Techniques outlined in “behavior change technique taxonomy” [32,33]; ^b^ Mediators outlined in the Social Cognitive Theory (SCT) [34] and Self-Determination Theory (SDT) [35].

**Table 2:** Baseline characteristics of Step it Up Families.

**Children**		**All**	**Girls**	**Boys**	***p* Value**
*n* (%)		58 (100.0)	29 (50.0)	29 (50.0)	
Age, Mean (*SD*)		8.0 (1.5)	8.1 (1.5)	7.8 (1.5)	0.491
MVPA min/day, Mean (*SD*)		56.1 (37.3)	61.5 (45.6)	50.6 (25.9)	0.271
Meeting physical activity guidelines, *n* (%)		19 (33.3)	10 (34.5)	9 (32.1)	0.851
**Parents**		**All**	**Mothers**	**Fathers**	***p* Value**
*n* (%)		72	39 (97.5)	33 (82.5)	
Age, Mean (*SD*)		39.3 (5.4)	37.8 (4.3)	41.2 (6.1)	0.010
Education, *n* (%)					0.217
	13+ years	30 (76.9)	51 (70.8)	21 (63.6)	
	0–12 years	9 (23.1)	21 (29.2)	12 (36.4)	
Work status, *n* (%)					<0.001
	Employed	29 (74.4)	59 (81.9)	30 (90.9)	
	Unemployed	10 (15.6)	13 (18.1)	3 (9.1)	
Ethnicity, *n* (%)					0.354
	Caucasian	38 (97.4)	71 (98.6)	33 (100.0)	
	Asian	1 (2.6)	1 (1.4)	0 (0.0)	
MVPA min/day, Mean (*SD*)		9.4 (11.7)	8.6 (9.8)	10.4 (13.8)	0.535
Meeting physical activity guidelines, *n* (%)		9 (12.7)	3 (7.7)	6 (18.8)	0.163

MVPA = moderate-to-vigorous physical activity, M = mean, *SD* = standard deviation.

**Table 3:** Pre-post Intervention Physical Activity Changes in Children, Mothers and Fathers.

Participants	Participants with Complete Data				Intention-to-Treat			
	*n*	Pre	Post	Difference ^a^	*N*	Pre	Post	Difference ^a^
**All children**								
MVPA min/day, Mean (*SD*), (95% CI)	44	51.7 (32.8)	109.5 (56.1)	+57.8 (40.5, 75.0)	57 ^b^	56.1 (37.3)	100.7 (56.3)	+44.6 (29.9, 59.3)
Meeting physical activity guidelines, *n* (%)	44	15 (34.1)	39 (88.6)	+24 (54.5)	57	19 (33.3)	43 (75.4)	+24 (42.1)
**Boys**								
MVPA min/day, Mean (*SD*), (95% CI)	23	49.9 (28.5)	104.0 (46.8)	+54.0 (33.9, 74.2)	28 ^b^	50.6 (25.9)	95.0 (46.6)	+44.4 (26.2, 62.6)
Meeting physical activity guidelines, *n* (%)	23	8 (34.8)	21 (91.3)	+13 (56.5)	28 ^b^	9 (32.1)	22 (78.6)	+13 (46.5)
**Girls**								
MVPA min/day, Mean (*SD*), (95% CI)	21	53.7 (37.6)	115.5 (65.4)	+61.8 (31.2, 92.5)	29	61.5 (45.6)	106.3 (64.7)	+44.8 (20.6, 68.9)
Meeting physical activity guidelines, *n* (%)	21	7 (33.3)	18 (85.7)	+11 (52.4)	29	10 (34.5)	21 (72.4)	+11 (37.9)
**All parents**								
MVPA min/day, Mean (*SD*), (95% CI)	65	9.6 (12.1)	38.3 (33.6)	+28.7 (20.4, 37.1)	71^b^	9.4 (11.7)	35.7 (33.3)	+26.3 (18.4, 34.1)
Meeting physical activity guidelines, *n* (%)	65	9 (13.8)	40 (61.5)	+31 (47.7)	71^b^	9 (12.7)	40 (56.3)	+31 (43.6)
**Mothers**								
MVPA min/day, Mean (*SD*), (95% CI)	37	8.5 (10.0)	35.8 (35.6)	+27.3 (15.6, 39.0)	39	8.6 (9.8)	34.6 (35.1)	+25.9 (14.7, 37.2)
Meeting physical activity guidelines, *n* (%)	37	3 (8.1)	21 (56.8)	+18 (48.7)	39	3 (7.7)	21 (53.8)	+18 (46.1)
**Fathers**								
MVPA min/day, Mean (*SD*), (95% CI)	28	11.0 (14.5)	41.6 (31.1)	+30.5 (18.0, 43.0)	32 ^b^	10.4 (13.8)	37.1 (31.5)	+26.7 (15.3, 38.2)
Meeting physical activity guidelines, *n* (%)	28	6 (21.4)	19 (67.9)	+13 (46.5)	32 ^b^	6 (18.8)	19 (59.4)	+13 (40.6)

Abbreviations: MVPA = moderate-to-vigorous physical activity, *SD* = standard deviation; ^a^ All pre-post intervention differences were significant at *p* < 0.001 in either analysis approach: participants with complete data and intention-to-treat.; ^b^ One participant had missing physical activity data at baseline and therefore was excluded from the intention-to-treat analysis.

## Supplementary Materials

The following are available online at https://www.mdpi.com/1660-4601/17/20/7655/s1, Supplementary File S1: Intervention materials.

## Abbreviations

AAS: Active Australia Survey; CLASS: Children’s Leisure Activity Study Survey; ICC: intraclass correlation coefficient; M: mean; MVPA: moderate-to-vigorous physical activity; SCT: social cognitive theory; *SD*: standard deviation; SDT: self-determination theory.
