# Supplementary material for: Effects of an Activity Tracker and App Intervention to Increase Physical Activity in Whole Families—The Step It Up Family Feasibility Study
Source: Int J Environ Res Public Health. 2020 Oct 20;17(20):7655. doi: 10.3390/ijerph17207655 (PMC7588994; doi:10.3390/ijerph17207655)
Supplement: Supplementary file 1 [file ijerph-17-07655-s001.pdf]

Supplementary File 1

Intervention materials

Children and both parents were instructed (during the introductory session) to use these features in the activity trackers and apps for children and parents for implementing these intervention components:

- Setting individual and family goals
- Setting graded achievable goals
- Self-monitoring
- Family leaderboard (displayed in Vivofit Jr app for children and also in the Family Step Challenge Log Poster)
- Family step challenges

Children and parents were free to decide when and to what extend they wanted to implement these intervention components/behavior changes techniques and utilise these activity tracker and app features during the 6-week intervention. Apart from the introductory session conducted at the start of the intervention, families received no further instructions by the research team on how to use the activity tracker and app features.

Garmin Vivofit Jr activity tracker for children

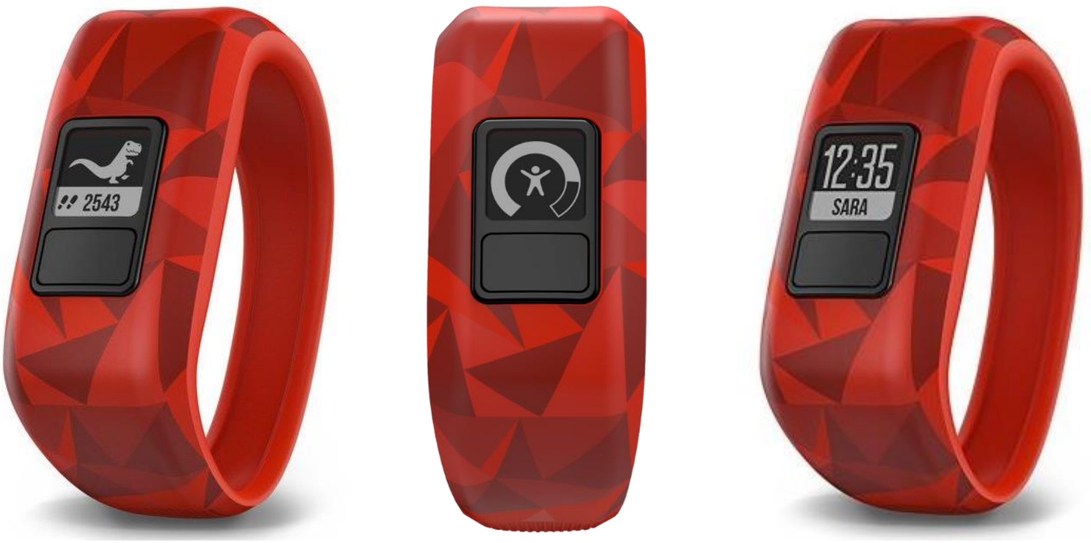

Note: For children, only the red activity tracker band was used to be gender-neutral and avoid arguments between siblings regarding colour choice. If the red band Garmin designed for children was too tight on the wrist of a child, the child could choose between one of the adult bands (white or black colour, see below). As the activity trackers were set up with children's names on display, siblings could distinguish between their activity trackers.

Garmin Vivofit 3 activity tracker for parents

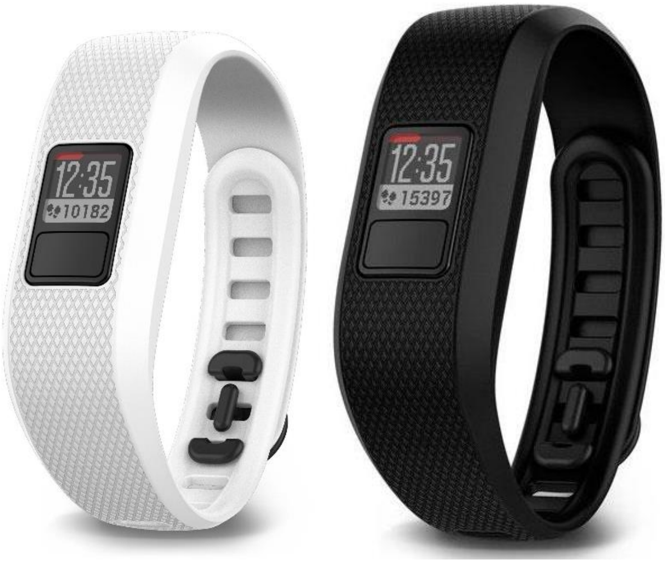

Note: Parents were offered the choice between a white or black coloured band to make it easier for parents and children to distinguish between the activity tracker of Mum and Dad. Unlike the Vivofit Jr activity tracker, the Vivofit 3 activity tracker for adults did not show names on the display.

Garmin Vivofit Jr app for children

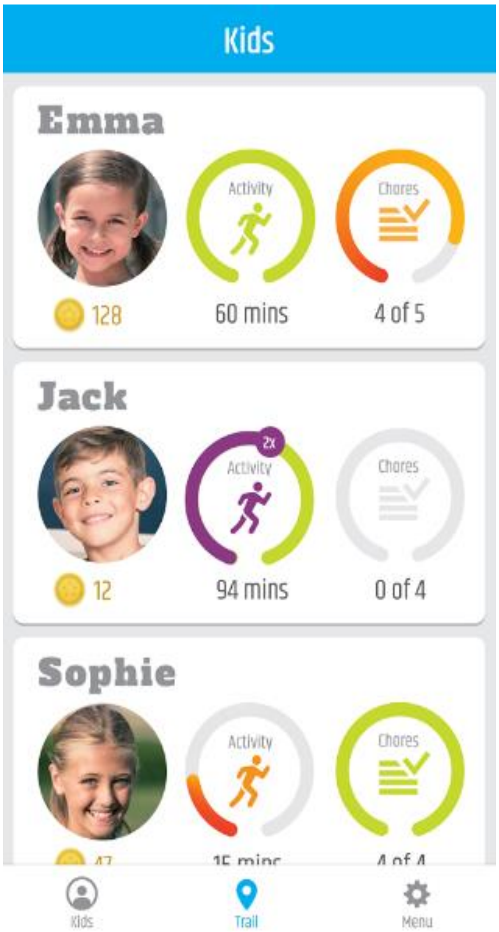

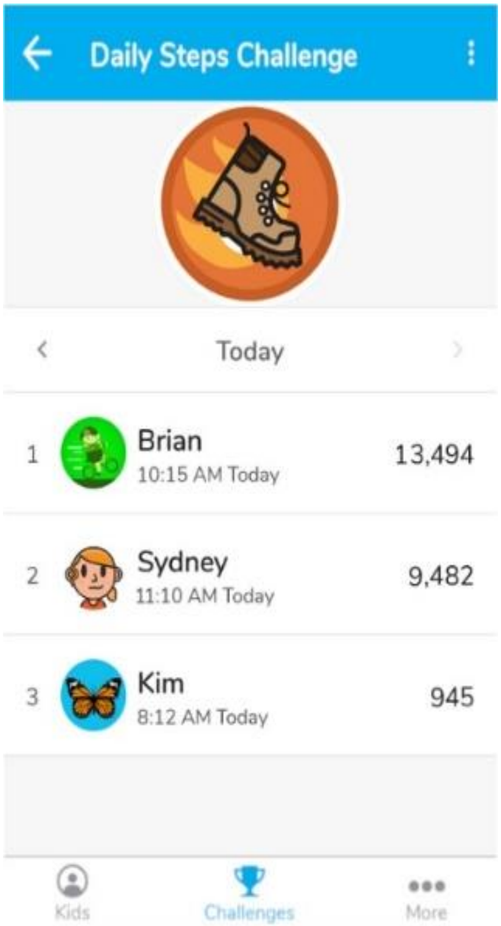

Children were instructed to use the '60 Minutes Activity' and 'Daily Steps Challenge' features in the app displays.

Garmin Connect app for parents

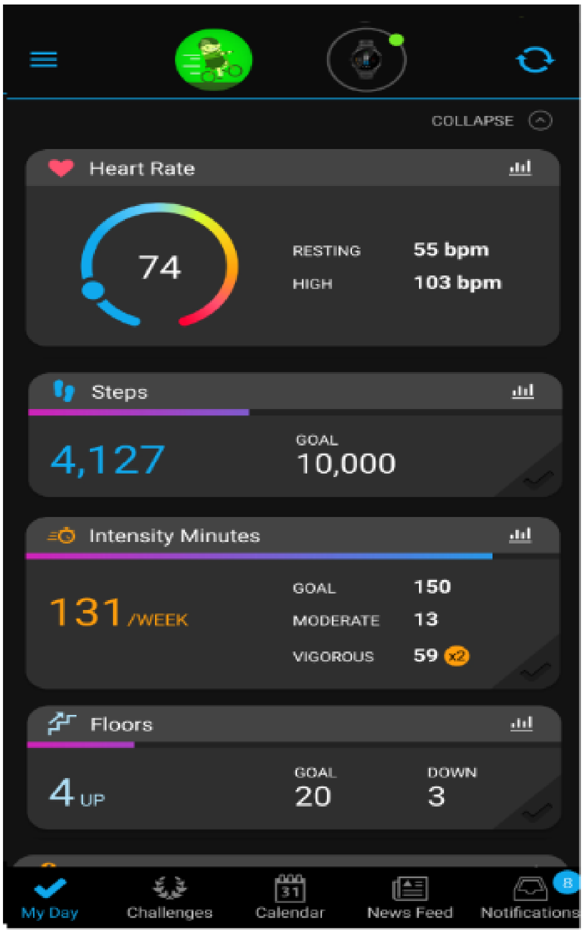

Parents were instructed to use the 'Intensity Minutes' and 'Steps' features in the app display.

Family Step Challenge Log poster

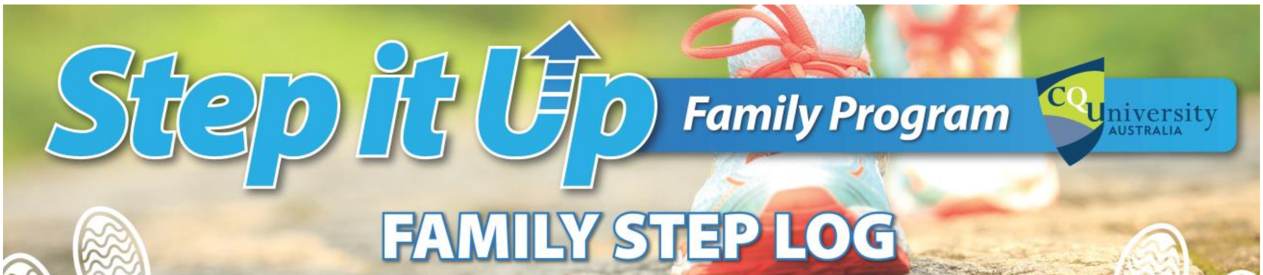

EXAMPLE WEEK - DATE	2 / 5 / 17	3 / 5 / 17	4 / 5 / 17	5 / 5 / 17	6 / 5 / 17	7 / 5 / 17	8 / 5 / 17	INDIVIDUAL TOTAL
MUM	4,166	5,367	6,839	10,267	9,377	10,627	9,334	55,977
DAD	5,245	6,678	7,987	8,743	9,405	10,789	8,732	57,579
BILLY	6,334	8,578	9,356	10,488	9,893	10,893	11,356	66,898
NINA	6,789	7,993	9,473	9,883	10,534	9,876	10,933	65,481
FAMILY TOTAL	22,534	28,616	33,655	39,381	39,209	42,185	40,355	
							EXAMPLE WEEK FAMILY TOTAL	245,935

EXAMPLE

WEEK 1 - DATE	/ /	/ /	/ /	/ /	/ /	/ /	/ /	INDIVIDUAL TOTAL
FAMILY TOTAL								
							WEEK 1 FAMILY TOTAL	

WEEK 2 - DATE	/ /	/ /	/ /	/ /	/ /	/ /	/ /	INDIVIDUAL TOTAL
FAMILY TOTAL								
							WEEK 2 FAMILY TOTAL	

WEEK 3 - DATE	/ /	/ /	/ /	/ /	/ /	/ /	/ /	INDIVIDUAL TOTAL
FAMILY TOTAL								
							WEEK 3 FAMILY TOTAL	

WEEK 4 - DATE	/ /	/ /	/ /	/ /	/ /	/ /	/ /	INDIVIDUAL TOTAL
FAMILY TOTAL								
							WEEK 4 FAMILY TOTAL	

WEEK 5 - DATE	/ /	/ /	/ /	/ /	/ /	/ /	/ /	INDIVIDUAL TOTAL
FAMILY TOTAL								
							WEEK 5 FAMILY TOTAL	

WEEK 6 - DATE	/ /	/ /	/ /	/ /	/ /	/ /	/ /	INDIVIDUAL TOTAL
FAMILY TOTAL								
							WEEK 6 FAMILY TOTAL	

Text messages sent 3x per week to parents' smartphone

Mainly practical, motivational behaviour-based and some knowledge-based mobile phone text messages! Tips and hints

When to send	Characters with spaces (max 160)	Behaviour change technique	Text message content
Week 0	160		Hello Step it Up Family! Over the next 6 weeks I will give your family tips on how to become more active. Contact Deb for help: 49232040 or stepitup@cqu.edu.au
Week 1	160	Prompt intentions formation<br>Set graded tasks<br>Prompt specific goal setting<br>Prompt practice	10,000 steps/day is a great goal for each family member. Start low, gradually build up. Check todays steps, add 500 steps tomorrow! Feels great to reach goals 😀
Week 1	159	Prompt intentions formation<br>Set graded tasks<br>Prompt specific goal setting<br>Prompt practice	30,000 Steps/day is a great goal for a family of 3. Start with small family step goals (e.g. 1,500 steps more each day) and develop ideas for step activities! 😇
Week 1	158	Prompt practice<br>Provide instruction<br>Provide general encouragement<br>Prompt identification as role model	Feeling a bit rusty? Get steps by practicing skills like hopping, jumping, throwing, and catching a ball. Show your kids how it's done - or let them show you! 😀
Week 2	158	Prompt intentions formation<br>Set graded tasks<br>Prompt specific goal setting<br>Prompt practice	Is your family easily reaching their step goals? Maybe it's time to 'step it up' – add 1,000 more steps/day this week if it's becoming too easy. 💪
Week 2	159	Prompt intentions formation<br>Set graded tasks<br>Prompt specific goal setting<br>Provide instruction<br>Prompt practice	60 min/day activity for your kids includes walking/cycling to places, outdoor play in the garden, or organised sports. Replace screen time with active time! 😀
Week 2	155	Prompt practice<br>Prompt identification as role model	Mums and Dads: Children LOVE playing active games with their parents. Look up how to play 'Kick the can' or 'Flag Footy'
Week 3	148	Provide general encouragement<br>Prompt practice<br>Plan social support	Congratulate your kids 👍 when they meet their daily step goals and every time they beat their own daily best, it will motivate them to keep stepping! 👣
Week 3	150	Prompt intentions formation	No time to be active? Find your steps in everyday life: park the car a bit further away,

		Prompt specific goal setting<br>Provide instruction<br>Prompt practice	walk around when on the phone, clean the house with music on! 🎵😀
Week 3	161	Provide information about behaviour-health link<br>Provide general encouragement<br>Prompt identification as role model	Fact: Rough-and-tumble play is a good activity for the confidence, resilience and emotional control of your kids. Keep it fun and let them win from time to time 😀
Week 4	160	Prompt intentions formation<br>Provide instruction<br>Prompt practice<br>Provide general encouragement	Active kids are better at doing homework and often do better at school! Encourage outdoor play to release energy, so they can concentrate better afterwards. 🏃‍♀️🏃‍♂️🤓
Week 4	158	Provide general encouragement<br>Prompt practice<br>Provide instruction<br>Plan social support	Kids: Coach Mum and Dad to be more active! Make an obstacle course or play footy 🏉⚽ in the garden with Mum or Dad, or take them for a walk in the neighbourhood.
Week 4	155	Prompt practice<br>Provide instruction<br>Plan social support<br>Prompt identification as role model	Boost your steps with family walks on the weekend: do beach walks, walk up the Bluff, the Rail Trail or even Mt Archer if you dare!
Week 5	122	Provide general encouragement<br>Plan social support	Be active together with your kids and do something everybody likes! If you're having fun, you will keep it up for longer. 😀
Week 5	155	Plan social support	Don't know what activities to do? Ask your kids what they like doing best…I'm sure they will have lots of creative ideas to get the whole family going! 😀
Week 5	143	Prompt practice<br>Provide instruction<br>Plan social support<br>Prompt identification as role model	Actions speak loader than words: Model an active lifestyle to your kids by being active at home or whilst watching them at sporting activities.
Week 6	117	Plan social support<br>Provide instruction<br>Provide general encouragement	Teach your kids to play classics like hopscotch, tag, or hide-and-seeks. And then join in so you get more steps too! 😀👣
Week 6	151	Prompt practice<br>Provide instruction<br>Prompt identification as role model	Create active family routines such as regularly walking to the shops, taking the dog to the park or washing the car together as a family.
Week 6	136		Hello Step it Up Family! This is the end of the Step it Up Family Program. We hope you had fun! We'll be in touch to get your feedback 😀
